# The importance of creating the right conditions for group intervision sessions among medical residents– a qualitative study

**DOI:** 10.1186/s12909-024-05342-0

**Published:** 2024-04-05

**Authors:** Anouk Jorissen, Kim van de Kant, Habibe Ikiz, Valerie van den Eertwegh, Walther van Mook, Angelique de Rijk

**Affiliations:** 1 Department of Social Medicine, Care and Public Health, Research Institute (CAPHRI), Maastricht University, Maastricht, the Netherlands; 2 Academy for Postgraduate Medical Training, Maastricht University Medical Center, PO Box 5800, Maastricht, 6202 AZ the Netherlands; 3 Department of Family Medicine, Care and Public Health, Research Institute (CAPHRI), Maastricht University, PO Box 5800, Maastricht, 6202 AZ the Netherlands; 4 Department of Gynecology, Maastricht University Medical Center, Maastricht, the Netherlands; 5 Skillslab, Faculty of Health, Medicine and Life Sciences, Maastricht University, Maastricht, the Netherlands; 6 Intensive Care Medicine, Maastricht University Medical Center, Maastricht, the Netherlands; 7 School of Health Professions Education (SHE), Maastricht University, Maastricht, the Netherlands

**Keywords:** Group reflection sessions, Personal resources, Burn-out, Medical doctors

## Abstract

**Background:**

The burnout rates among residents urge for adequate interventions to improve resilience and prevent burnout. Peer reflection, also called group intervision sessions, is a potentially successful intervention to increase the resilience of young doctors. We aimed to gain insight into the perceived added value of intervision sessions and the prerequisite conditions to achieve this, according to residents and intervisors. Our insights might be of help to those who think of implementing intervision sessions in their institution.

**Methods:**

An explorative, qualitative study was performed using focus groups and semi-structured interviews with both residents (*n* = 8) and intervisors (*n* = 6) who participated in intervision sessions in a university medical center in the Netherlands. The topic list included the perceived added value of intervision sessions and factors contributing to that. The interviews were transcribed verbatim and coded using NVivo. Thematic analysis was subsequently performed.

**Results:**

According to residents and intervisors, intervision sessions contributed to personal and professional identity development; improving collegiality; and preventing burn-out. Whether these added values were experienced, depended on: (1) choices made during preparation (intervisor choice, organizational prerequisites, group composition, workload); (2) conditions of the intervision sessions (safety, depth, role of intervisor, group dynamics, pre-existent development); and (3) the hospital climate.

**Conclusions:**

Intervision sessions are perceived to be of added value to the identity development of medical residents and to prevent becoming burned out. This article gives insight in conditions necessary to reach the added value of intervision sessions. Optimizing preparation, meeting prerequisite conditions, and establishing a stimulating hospital climate are regarded as key to achieve this.

## Background

During their professional training as medical specialist, residents experience exponential levels of professional and personal growth. This development can however be challenging. High levels of burnout among residents are widely reported, ranging from 25% to > 75%, depending on specialty [1–5]. Burnout is characterized by severe exhaustion, mental distance, emotional deregulation, and cognitive deregulation [6]. Consequences, such as depression, substance abuse, suboptimal patient care and limited productivity, are alarming and have a negative impact on residents themselves [7–11]. Further, the social environment suffers from the displayed lack of empathy and unexpected emotional eruptions, the quality of patient care reduces due to mental distance, and threatens to patient safety [1, 12–14].

The existing high levels of burnout have led to several attempts for reducing burnout, however consensus on the effective strategies is lacking. Various interventions proved to be effective including person-directed interventions (e.g. stress management, improving coping skills, cognitive behavioral therapy), group-directed interventions (e.g. intervision sessions), and organization-directed interventions (e.g. job training/education, training supervisors in recognition of symptoms) [15–17].

An effective intervention should address determinants of burnout. Burnout is generally determined by too high job demands (having to work too fast and too long) in the context of lack of resources to face these demands, as theorized by the the Job Demands Resources (JDR) model. Additionally, empirical research shows that resources have a direct positive effect on workers’ wellbeing, such as decreasing burnout [18, 19]. Residents have high demands that cannot be easily reduced directly. Thus improving resources is key. The existing high levels of burnout, especially among residents, have indeed led to attempts to improve residents’ personal and job-related resources [19, 20]. Improving residents’ personal resources, can work in two directions. Residents’ self-confidence to dare to ask for improvements in psychosocial working conditions such as decreasing work demands can be given a boost. Secondly, personal and professional growth might be increased. Resilience, “the personal qualities that enable a person to adapt well and even thrive in the face of adversity and stress”, is regarded an important personal resource to “navigate the demands of professional live” and reduce burnout [21]. A potential successful intervention to improve residents’ personal resources during the stressful and exhausting period of residency is peer reflection sessions, also called group intervision sessions [22–24]. Intervision sessions are increasingly recommended to reinforce personal and job resources in medical education, and might thereby impact the level of burnout in medical students and residents [25, 26]. However, the potential added value of intervision sessions in residents has not yet been fully clarified. Moreover, the prerequisites (e.g. with respect to organization and content) to make intervision sessions a success have so far not been explored in residents. During group intervision sessions, residents have the opportunity to share, discuss and reflect on work-related experiences and learning moments. The aim of intervision sessions is to support residents’ personal- and professional development by using work-related situations as a starting point for reflection and discussion. Residents are stimulated to reflect on their own actions and emotions with challenging situations, as well as reflection on the experiences of others. Intervision sessions commonly take place within a group of residents, under the guidance of an intervisor (coach) to stimulate a safe and constructive climate. Previous studies showed that intervision sessions can result in improving one’s ability to produce a wider range of solutions for challenging professional situations, increasing self-management, and offering residents acknowledgment and (self-)confidence [27, 28]. Furthermore, intervision sessions can be helpful to identify individual resources and recovery-related self-efficacy [29]. Besides, these peer reflection sessions can improve group dynamics regarding respect and trust between residents, thereby stimulating a safe learning environment [30–33].

Evidence on how intervision sessions can successfully be incorporated in residency, and evidence regarding its effects is still limited. There are, for example, various methods and formats used for group intervision sessions, and it is unknown which factors contribute to making intervision sessions successful from the perspective of residents and intervisors.

The current explorative study therefore focuses on the perceived experiences of intervision sessions for medical residents. The aim of this study is to gain insight into the perceived added value of intervision sessions and the prerequisites to achieve this added value. Therefore, the following research questions were formulated: (1) How are group intervision sessions perceived and valued by residents and intervisors? (2) What conditions contributed to the value and quality according to residents and intervisors?

These insights have the potential to help to improve intervision sessions for residents as an effective method to increase their personal resources and decrease burnout rates.

## Methods

### Design in short

An explorative, qualitative study was performed using focus groups and semi-structured individual interviews with residents and intervisors from one University Medical Centre in the Netherlands. Structured questionnaires were used to collect background characteristics on residents, intervisors, and the intervision sessions.

### Study samples

#### Residents

In the period 2018–2020, about 150 residents participated in intervision sessions in the Maastricht University Medical Center + . Residents who participated in intervision sessions were approached via their residency program director, general online newsletters, colleague residents, and the academy of the university medical center. Upon expression of interest in participation, residents were sent a hyperlink to fill out contact information and some baseline characteristics (such as medical specialty). After providing additional information, informed consent could be given via a separate digital hyperlink. After informed consent, focus groups and individual interviews were planned and conducted. Focus groups were scheduled, and if impossible, individual interviews were planned. The purpose of the research, namely that we aimed to evaluate and improve intervision sessions, was explained to residents. Residents were stimulated to be open and honest on their experiences and opinions. Residents were ensured that the data were handled confidentially**.**

#### Intervisors

Intervisors who moderated the intervision sessions of residents from 2018 to 2020 at the Maastricht University Medical Center + were informed and invited. The intervisors included coaches and medical doctors, who were not involved in the medical education of residents and thereby had an independent relationship to the residents. After informed consent, a short questionnaire on the characteristics of the intervision groups (e.g. frequency, duration, number of participants) was filled out by the intervisors.

#### Data collection

Due to COVID-19 restrictions, all interviews/focus groups with residents and intervisors were held online, and participants were asked to participate with their microphones and video to optimize participation [34]. Both the focus group interviews and individual interviews were guided by an experienced moderator (VE) [35]. And an additional observer focused on non-verbal behavior, interactions, and group dynamics (HI, AJ). Before the focus groups, a topic list (see Table 1) was constructed by the research team (AR, KK, AJ) to guide the focus groups/interviews. For the intervisors, the above-mentioned questionnaire was followed by an online semi-structured individual interview. For the intervisors, focus groups were not chosen to avoid bias and socially desirable answers. All interviews were held by experienced interviewers. The topic list was comparable to that of the residents and could be used as flexible guidance for the moderator. Six interviews were held, and the content/list of topics discussed can be found in Table 1. The item-design for the topic list was informed by the research questions, the content of the intervision sessions, literature (on the JDR Model, and resources as resilience, coping and recovery) and subsequent discussion with various experts on intervision and coaching [19, 20, 36, 37].

**Table 1:** Topic list

Topic
Introduction and background of participation
The experienced added value of intervision sessions
Major events that residents perceived and how this was discussed during intervision sessions
To what extent and how intervision sessions contributed to resilience
What contributed to the quality of intervision sessions
Questions/remarks about the topics handled during intervision sessions

### Data analysis

Data from the online focus groups and individual interviews were anonymized and transcribed ad verbatim. Thematic analysis and the Quagol approach to analysis were applied [38], using NVivo 12 software©. The researchers started with ‘bracketing’ to control prejudice. Next, interviews were read line-by-line by AJ and AR Memos to express the essential elements of each interview were written and discussed to deepen the analysis. Next, initial coding took place and was discussed within the research team (AR, KK, AJ), to generate themes and subthemes. Further, relationships between the (sub)themes were analyzed using techniques such as analytical induction and were discussed (axial coding). The analysis started with the first two focus groups. During the coding process, all researchers were asked to critically review the (sub)themes and their relationship. The analysis found place until consensus was reached on the final themes and conceptual model. Data and themes were compared and contrasted. Through discussion, differences in interpretation were resolved until consensus was reached. Data collection was seized when thematic sufficiency was reached [39]. As suggested by literature, in this qualitative, exploratory study we strived for thematic sufficiency (instead of data saturation) [40]. Thereby we focused on the quality (not quantity) of the interviews and focus groups to generate rich data and new insights. We seized data collection when having ‘heard enough’ instead of having ‘heard it all’.

### Ethical considerations

This research was approved by the Netherlands Association of Medical Education (NVMO) Ethical Review Board (ID number: 2020.5.6). All residents and intervisors gave digital informed consent. Information that could be traced to the person was only available to the interviewer who handled it confidentially. Anonymization of all data and interviews took place during transcription.

### Scientific accuracy and reliability

The following strategies were used to assure reliability and accuracy: 1) Bracketing took place and a logbook was kept; [38, 41] 2) Member checks took place for every participant after each focus group [38, 42]. Based on the data collected, a new conceptual model was developed [38]. Timely moments of peer debriefing, with members and non-members of the research team, took place to discuss the methods and analysis [43]. A presentation within the research team was given with a discussion afterwards; intervisors also participated to improve the validity.

### Reflexivity

All authors have extensive expertise and affinity in the field of medical education and/or health psychology. AJ is MD and was the principal researcher. She initiated the design of the topic list, and selected the sample. Together with VE she performed the interviews. AJ and VE knew none of the participants and had no professional relationship with them. AJ transcribed the interviews and analyzed the data in collaboration with AR and KK.

KK has a PhD in epidemiology and is an assistant professor at the medical faculty and Academy for Postgraduate Medical Training at the Maastricht University Medical Center + . She coordinates a well-being program for residents. Together with AR and WM she initiated the research. She supervised AJ and contributed to the writing of the manuscript.

HI is MD and reviewed the literature and contributed to writing the manuscript.

VE has a PhD in communication skills training and transformational learning and is a course developer and trainer in communication and behavioral change programs at the Skillslab department of Maastricht University. When executing the interviews, she was coach in the intervision program in the master’s in medicine at Maastricht University.

WM is MD and has a PhD in professional behavioral development among doctors (in training). In addition to his work as an intensivist, he is professor of Professional Development, and director of the Academy for Postgraduate Medical Training. Next to initiating the study, he contributed to writing the manuscript.

AR has a PhD in health psychology and is a professor in Work and Health at the Department of Social Medicine. She initiated the research together with KK and WM and supervised every step of the research. During the research, she trained AJ regarding interviewing and qualitative data analysis. AR knew none of the participants and had no professional relationship with them.

## Results

### Baseline characteristics

In Table 2 and Table 3, the resident characteristics and characteristics of the intervention groups are provided, respectively. In Table 4, the intervisors’ characteristics are noted.

**Table 2:** Resident characteristics

Characteristics	Participants focus groups (*n* = 6)	Participants Individual interviews (*n* = 2)
Gender (number)		
Male	1	1
Female	5	1
Specialism (number)		
Internal Medicine	3	2
Pediatrics	2	0
Gynecology	1	0

**Table 3:** Characteristics of the intervision groups

Characteristics of the intervision groups (in numbers)	*n* = 6
Individual intakes	2
Participation mandatory	4
Intervision during working time	6
Duration of each intervision session in minutes > 120	6
Course of the intervision in months > 12	5
Number of participants 5–10 / > 10	4 / 2

**Table 4:** Intervisor characteristics

Intervisors characteristics	*n* = 6
Gender (number)	
Male	1
Female	5
Background (number)	
Medicine	3
HR-management	2
Psychology	1

**Fig. 1:**
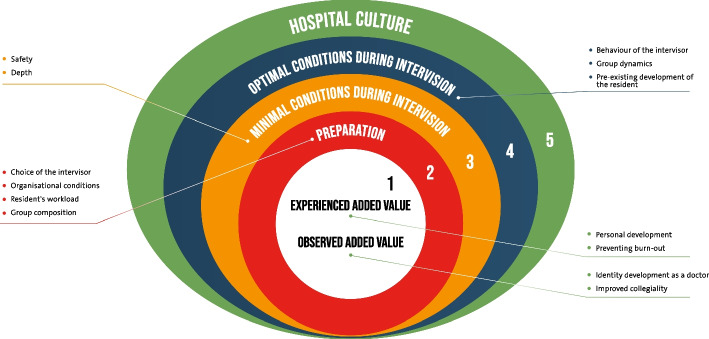
Conceptual model of the layers of factors contributing to the added value of intervision

Generally, both residents and intervisors perceived that the intervision sessions had varying levels of added value. Figure 1 offers the conceptual model of the factors that contributed to the added value. The level of added value depended on three types of factors: (1) choices already made during preparation, (2) conditions of the intervision sessions (both related to prerequisites and the process), and finally, (3) the hospital climate. First, the added value will be described, and the three types of factors. Finally, the interrelatedness between the factors will be addressed.

#### Factor 1: Added value

Residents described the intervision sessions contributed to their personal development and secondly, prevented becoming burned out. The intervisors perceived that the intervision sessions contributed to the development of the resident’s identity as a doctor and to the collegiality.

According to the residents, the intervision sessions contributed to personal development if they allowed residents to focus on their personal growth. The intervision sessions allowed them to learn about themselves and offered a moment for self-reflection. Moreover, hearing the struggles of other residents helped them to put things into perspective and improved their self-acceptation.

‘So nice to hear others, who are going through the same and then you think, it is not just me, it happens to others too.’ (Resident, R)

Because of sharing their struggles with e.g. work-related issues, residents experienced more liberty to let go of some problems and put themselves less under pressure. They felt more freedom to be vulnerable, especially about situations they were hesitant to discuss in their regular work due to the hospital’s closed social climate and hierarchal structure. The intervisors acknowledged that residents learned from each other.

‘The fact that you see other residents doing it, giving examples, offers you alternatives according to me.’ (R)

Further, residents emphasized that both having good contact with their intervisors and having more experienced residents in their intervision group were essential for the improvement of their self-awareness and -acceptance.

‘It is pleasant to see how an elder resident reflects on how it was for them in the first year, and how you grow.’ (R)

Secondly, intervision sessions were perceived as useful to prevent burn-out because they taught how to early recognize symptoms and confronted the residents with early burn-out symptoms in colleague residents.

‘You recognize faster that you’re in a certain situation and don’t let it get out of hand for yourself or the entire situation.’ (R)

Residents noted that intervision sessions increased resilience by focusing on how to recover and recharge while keeping in touch with what was considered important and made one happy. This was also acknowledged by the intervisors.

The intervisors acknowledged two additional added values. First, the development of their identity as a doctor, that is, ‘how to fill one’s white coat’.

Second, they also observed improvement in collegiality and sense of belonging.

‘We hear back from them that, at least, it strengthens their group cohesion.’ (Intervisor, I)

#### Factor 2: How added value can be achieved: Preparation

To achieve these four added values, some important choices had to be made in the preparation of intervision sessions according to the residents. This preparation can be regarded to first layer of subfactors affecting the added value.

First, the choice of the intervisor: according to the residents, the intervisor needs to express a human approach, including empathy and honesty. Hospital supervisors should not be involved, as it would compromise the aspect of safety (see below). Residents described that the intervisor does not necessarily need to have a medical degree.

Second, organizational conditions (such as frequency, location, and timing) of intervision sessions play a role. The optimal frequency of sessions varied between residents, with both positive and negative experiences noted for high session frequency (e.g. every 6 weeks vs. every 3 months). High frequency was linked to increased comfort and depth of the discussions by the residents. However, residents also noted that they preferred longer-lasting trajectories of intervision instead of a booster for a shorter period. In addition, longer episodes of intervision sessions (during their whole residency) were perceived as having a stronger effect on their personal development. The intervisors agreed on an optimal frequency of once in six weeks without preferring boosters or long-term episodes. Concerning the location for the session: A room within the hospital was preferred to avoid travel time. The room should have a good temperature and some daylight. Snacks were considered to contribute to a relaxed atmosphere.

With respect to the timing of intervision sessions, residents preferred that interference with clinical duties to be avoided, to allow them to fully focus on themselves and the intervision sessions. The residents’ workload should not be increased by the intervision sessions. Both residents and intervisors pled for sessions during work hours.

Residents and intervisors also agreed that sessions should take about 2.5 to three hours, giving enough time to acclimatize, create safety, and allow in-depth discussions.

‘It is just, not a lot of people have enough time for it. You have 10 min and then your pager goes. Yeah, it’s just, the moments of rest are too little.’ (R)

To increase the level of depth during intervision sessions, an individual interview between the intervisor and residents *before* the start of the intervision sessions was considered a significant advantage by both parties. During this interview, personal learning goals could be formulated to facilitate in-depth reflections and more fine-tuned personal development. Additionally, the interviews were reported to contribute to safety because of the opportunity to ventilate expectations and set boundaries on what (not) to discuss during intervision sessions.

‘And in the individual interview, I try to get the learning question/goal clear. One that goes deeper than; “I want to discuss better with my supervisor” or “I want a better work-life balance”….. I always seek what lies underneath these statements.’ (I).

Thirdly, residents described group composition as important. As long as the composition allowed for an atmosphere of confidence, it did not matter to residents whether the group comprised of colleagues they already knew, or of new residents from another or the same specialty. Rather, the continuity of the group composition throughout the process was regarded as an important condition.

Both residents and intervisors agreed that the optimal group size was five to seven residents, to create safety and depth.

‘It also helped to have smaller groups, people you knew and daring to be vulnerable.’ (I)

#### Factor 3: How added value can be achieved: Minimal conditions for intervision sessions itself

In addition to preparation, the minimal conditions constituted a second layer that affected added value. Residents described two prerequisites, and minimal conditions to experience the added value of intervision sessions: safety and depth (see Fig. 1).

Safety was described by residents as essential, salient, and indispensable.

‘There was an atmosphere that felt like everything being said here, is safe, and is not discussed outside the group. I think that is very important, to be honest, and vulnerable.’(R).

The role of the intervisor was reported to create such a safe environment by setting boundaries while also providing the residents with the liberty to keep control. This was described to aid in opening up and showing vulnerability both during intervision sessions as well as in the hospital setting. This resulted in getting to know each other better and understanding among each other why colleagues behave the way they behave.

‘But I think that in cultures such as in the hospital culture, which is not open, it is difficult to show your vulnerabilities. At least in intervision sessions, exactly this point can play a very big role.’ (R)

The second prerequisite, depth, was defined by residents as stimulating to think and listen to each other and was considered essential in intervision sessions. By sharing daily and complex situations encountered at work or home, residents mentioned they could ventilate their emotions, gained a perspective on how to adapt behaviour and facilitate providing feedback. Similarly, intervisors emphasized that in-depth discussions were essential for intervision sessions. They defined depth as making the translation to yourself and digging deeper into situations or problems. This depth fuelled the intervision sessions to go beyond the level of “only complaining and not changing”.

‘It was never superficial. The time of complaining quickly passed, and then we went deeper, and that in my opinion is what gave added value.’ (R)

#### Factor 4: How added value can be achieved: Conditions for optimizing intervision sessions

Fulfilling the aforementioned prerequisites did not lead to good intervision sessions by itself, rather they facilitated more intensive interaction between the intervisor and residents. Three factors that affected this intensity, and thereby maximized the effects of intervision sessions according to both residents and intervisors, were mentioned: the behaviour of the intervisor, the group dynamics, and the pre-existing development of the resident (See Fig. 1).

First, the behaviour of the intervisor had an important role in achieving increasing self-awareness. The working methods used by the intervisors were often customised. The intervisors had in common that they all tried to be active and create a safe environment. They aimed to allow residents to let go of the hierarchal hospital culture, allowing them to work on their insecurities and personal issues. Both the residents and intervisors reported the importance of enthusiasm and personal recognition shown by the intervisors so that residents were empowered.

‘I think that the intervisor revealed more in certain persons than someone who wasn’t trained to do this.’ (R)

Residents described that a good intervisor behaviour included noticing the non-verbal signals, digging deeper into experiences, and stimulating and monitoring the group process.

‘I asked them why you are so proud of that, can you tell us more about that, and why that happened. Then you see them shining.’ (I)

Second, the dynamics of the group (including recognition, normalisation of feelings, openness, vulnerability, and trust) contributed to both a better recognition of daily struggles during intervision sessions with colleagues in the same phase of their residency, and to learn from residents who were in a different phase.

‘I found it interesting for myself, as a senior resident, to see how junior residents are asking and still busy searching. Then you see the growth you made yourself, and how new questions pop up.’ (R)

Third, the resident’s pre-existing self-awareness and development were described as accelerating the depth-reaching process. Some residents took individual coaching and reported the experience of a synergy regarding their personal development between the intervision sessions and individual coaching. They noted that individual coaching, which stimulated their self-awareness, helped them to profit more from the intervision sessions. In line with this, residents who experienced more work-life balance experienced also more room to profit from the contribution of intervision sessions to self-development.

#### Factor 5: How added value can be achieved: Hospital culture

Finally, the intervisors mentioned the negative effect of the hospital culture on all aspects of intervision sessions. A non-functional hierarchy can influence the residents’ personal and professional development negatively according to intervisors.

‘Realistically, it is true that in a team you should be able to say anything, everything should be able to be discussed. But the hierarchy in a hospital is difficult, yet different from the hierarchy in a ministry or business environment.' (I)

Moreover, residents frequently appear to be attached more to their white coats than to paying attention to their personal ‘being’. Intervisors regard it important that intervision sessions add to a healthier balance between thinking and feeling.

‘But a doctor has an identity attached to his white coat more than all other professionals.

If that coat is taken off, they won't suddenly fall off their pedestal, but you're in some kind of unknown territory.’ (I)

### Both views

In this study the perspectives of both residents and intervisors were incorporated. This allows for comparison, and triangulation of data between both groups. For example, compared to the residents, the interviors presented an extra perspective on the culture of the hospital with its hierarchy. Moreover, they stressed the importance of intervision session in professional identity development and promoting collegiality.

### Interrelatedness

Taken together, as illustrated in Fig. 1, in order to achieve the added value of intervision, four layers of factors should be addressed: paying attention to a proper preparation; meeting both minimal and optimal conditions during intervision sessions; and taking care of a safe and stimulating hospital culture. It should be noted that these layers affect each other mutually. For example, reaching depth in the discussion during intervision sessions depends on group dynamics, which in turn depends on hospital culture. In this regard the figure should not read as one-to-one relationship between the factors, but more as a dynamic interplay where each factor is depending on factors at (some) other layers in the system.

### Implementing intervision

**Fig. 2:**
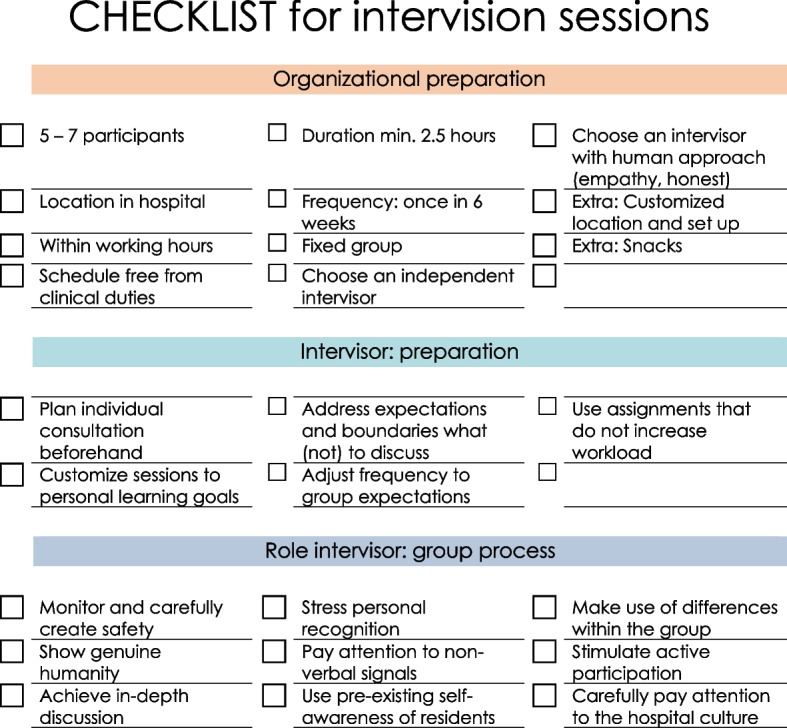
A practical checklist for incorporating intervision sessions into an institutions’ professional support program

To increase the practical usefulness of our research, we have summarized our findings in a practical guide and checklist for those interested in implementing intervision sessions themselves (Fig. 2).

## Discussion

The high burnout rates among residents urge for adequate interventions to improve residents’ personal resources and prevent burnout. The effects of intervision sessions are promising, but the number of studies on this topic is still small. This qualitative study seems to reveal that intervision can add value to residents’ personal and professional identity development, could improve collegiality, and might prevent burn-out. To achieve these added values, our findings point to considerable attention that should be paid to optimize preparation (intervisor choice, organisational prerequisites, group composition, workload), to meet the minimal and optimal prerequisite conditions of intervision sessions (safety, depth, role intervisor, group composition, pre-existent development), and to provide a hospital climate that stimulates personal and professional development.

### Improving resources to deal with job demands

There is increasing knowledge of the etiological factors of burn-out in residents [1, 14, 44]. The JDR Model [45] is a valuable and often cited model in this regard. It stresses the importance of balance between job demands and job resources [45]. Residents' work involves considerable job demands, such as long working hours, time pressure, increasing difficulty of tasks as trainees, and exposure to human suffering. Although reducing excessive demands is importance, not all of them can be eliminated easily, and system changes take time. Next to paying attention to job demands, according to the evidence for the JDR Model [29], both job resources and personal resources are directly related to lower burnout scores. Resilience is an important personal resource, and physicians have higher scores for resilience than the general population [21]. If the duration of exposure to excessive job demands is limited and the number of stressors is not too high, the residents can deal with them resiliently. In this regard, stressors could then also provide a positive challenge, an opportunity to learn from [46, 47]. Nevertheless, there might be room for improvement to increase resilience in residents, and effective interventions that boost personal-, and job-related resources, could empower residents to handle the substantial job demands they face. Moreover, improving personal resources, can increase personal and professional growth, and can empower residents to pro-actively improve work demands to a certain extent, making the knife cut both ways.

### The added value of intervision sessions: impact on resources

There is increasing evidence in the field of social work [28], nursing [48], and community practitioners [24], demonstrating that intervision sessions may aid in developing adequate coping strategies and strengthens one’s personal and professional development [31]. However, there is still limited understanding of the exact explanatory process on how intervision session contributes to residents’ coping and development. Our exploratory study, provides new insights into which factors contribute to the success of intervision sessions from the perspective of residents and intervisors.

The interviews did not reveal clear examples of reducing job demands or increasing job resources. The respondents tended to emphasize the increase of their personal resources, such as taking different views and self-esteem because of noticed personal growth. This study thus clearly shows that intervision sessions could be one of the promising interventions to boost the residents’ resources, thereby preventing burn-out symptoms. Intervision sessions enable to reflect in a safe environment on one's behavior, discuss it with colleagues, formulate behavioral alternatives, and thereby strengthen individual resilience. These positive experiences confirm previous research [22–24]. Besides, our results point at the added value of intervision sessions to personal and professional development. As a consequence, intervision sessions can help to better identify individual work-related and personal resources, and preferences for essential recovery and recharge [22–24, 49]. Moreover, this study revealed that intervision sessions can contribute to collegiality and a sense of belonging, an important job resource to alleviate burnout [50]. This is in line with previous studies reporting that peer reflection sessions can improve group dynamics regarding respect and trust between residents, thereby stimulating a safer daily learning environment in the long term [30–33, 51]. By encouraging residents to share their experiences, in a safe environment, they will learn from each other, e.g. concerning private-work balance, time management, and stress management [52, 53]. Previous literature confirms that intervision sessions aid in talking about work-related experiences and situations and contribute to safety and depth in individual self-reflection and personal well-being [28, 54]. The active ingredients of intervision thereby seem thus also be in line with the Self-Determination Theory (SDT) [55] next to the JDR Model. The SDT emphasizes the resources autonomy, competence, and relatedness to improve (employee) well-being. Van den Broeck et al. explain that the SDT model has an added value to the JDR model, as the SDT model additionally explains specific resources and the motivational process underlying burnout. Fulfilment of the three psychological needs expressed in the SDT represent important job-related resources [56]. Additionally, we found that the intervision in itself addressed these psychological needs.

### Implementing intervision: impacting factors on the experienced added value of intervision sessions

Our study emphasizes the impact of various factors on the experienced added value of intervision sessions, which should be considered when implementing intervision. Well-considered choices should be made on the choice of intervisor, organizational factors (including the intensity of intervision sessions), and group composition. Moreover, safety, depth, and no increased workload appeared prerequisites for successful intervision sessions. Besides, the role of the intervisor, a well-established group, and the pre-existent self-awareness of residents are important factors to maximize the effect of intervision sessions. Finally, our model emphasizes the impact of the hospital culture, often characterized by its high working pressure, overworked employees [1, 46], and hardworking mentality [57]. This stresses the importance of making efforts at organizational level, to improve the organization’s social climate. If employees do not see that there is a willingness on the part of staff members or management to make improvements in the hospital culture, the employees will become demotivated, and this will negate the added value of intervision sessions in the longer term. In this regard, we like to adhere to warnings made by other researchers when change is only asked at the individual level and not supported at the organizational level [58, 59].

### Limitations and further study

Some limitations of this study should be noted. First, we strived for a purposive sampling that captures the important characteristics of the study population. It should be noted that the size of the resident research population was limited, with an underrepresentation of male participants. Thereby gender specific factors could not have been studied. Several recruitment strategies were attempted having limited success, which is probably due to competing demands because of the COVID-19 care during our study. However, thematic sufficiency was acquired as we included both participants and intervisors, and data were analysed with rigor. Since our study is explorative in nature, we recommend future research to investigate the perceived value and prerequisite conditions on a larger scale. Second, our participants were recruited from one university hospital, making it worthwhile for further research in other hospital settings. In this regard, additional (quantitative and qualitative) research is needed to validate and enrich our model regarding the added value of intervision sessions.

## Conclusions

In conclusion, both residents and intervisors noted that intervision sessions can add value to the identity development of medical residents and can prevent burnout. However, before planning intervision sessions, careful decisions should be made concerning optimizing preparation, meeting the minimal and optimal prerequisite conditions, and establishing a stimulating hospital climate to achieve this.

Our findings are summarized and incorporated in a practical guideline: ‘Checklist for intervision sessions’ (Fig. 2), that can be of help for those who plan to implement intervision sessions in their institution.

## Data Availability

The dataset analyzed during the current study is available from the corresponding author on reasonable request.

## Abbreviations

I: Quote of Intervisor
R: Quote of Resident
